# Ten-years outcome analysis in patients with clinically localized prostate cancer treated by radical prostatectomy or external beam radiation therapy

**DOI:** 10.3389/fsurg.2022.966025

**Published:** 2022-07-29

**Authors:** Shu-Wen Li, Allen W. Chiu, Andy C. Huang, Yu-Wei Lai, Jyh-Der Leu, Yi-Chun Hsiao, Shiou-Sheng Chen, Thomas Y. Hsueh

**Affiliations:** ^1^ Division of Urology, Department of Surgery, Taipei City Hospital renai Branch, Taipei, Taiwan; ^2^ Department of Urology, School of Medicine, National Yang-Ming Chiao-Tung University, Taipei, Taiwan; ^3^ Division of Urology, Department of Surgery, Mackay Memorial Hospital, Taipei, Taiwan; ^4^ Division of Radiation Oncology, Taipei City Hospital Renai Branch, Taipei, Taiwan; ^5^ Division of Urology, Department of Surgery, Taipei City Hospital Zhongxiao Branch, Taipei, Taiwan

**Keywords:** intensity modulated radiotherapy, localized prostate cancer (PCa), laparoscopic radical prostatectomy, prostate cancer-specific survival, metastasis-free survival (MFS)

## Abstract

**Purpose:**

Since there was no consensus on treatment options for localized prostate cancer, we performed a retrospective study to compare the long-term survival benefit of radiotherapy (RT) versus laparoscopic radical prostatectomy (LRP) in Taiwan.

**Methods:**

218 patients with clinically localized prostate cancer treated between 2008 and 2017 (64 with LRP and 154 with RT) were enrolled in this study. The outcomes of RT and LRP were assessed after patients were stratified according to Gleason score, stage, and risk group. Crude survival, prostate cancer-specific survival, and metastasis-free survival were evaluated using the log-rank test.

**Results:**

The 5-year crude survival rate was 93.3% in the LRP group and 59.3% in the RT group. A significant survival benefit was found in the LRP group compared with the RT group (*p* = 0.004). Furthermore, significant differences were found in disease-specific survival (93.3% vs. 64.7%, *p* = 0.022) and metastasis-free survival (48% vs. 40.2%, *p* = 0.045) between the LRP and RT groups.

**Conclusions:**

Men with localized prostate cancer treated initially with LRP had a lower risk of prostate cancer-specific death and metastases compared with those treated with RT.

## Introduction

Prostate cancer is one of the most critical cancers worldwide and has the highest incidence among men in the United States (1). In Taiwan, prostate cancer is the fourth most commonly occurring cancer in men, and in 2016, it had the sixth highest cancer-related mortality rate (2). That year in Taiwan, an estimated 5,391 cases (0.023%) of prostate cancer were diagnosed and 1,347 men (0.006%) died of the disease. Several treatment modalities can be used for clinical localized prostate cancer, such as radiotherapy (RT), radical prostatectomy, or active surveillance, according to various clinical scenarios and the patient's preference. Since the 1990s, the development of laparoscopic surgery has resulted in rapid progress in managing localized prostate cancer. Several reports have evaluated different laparoscopic surgical techniques with favorable functional and oncological outcomes compared with conventional open radical prostatectomy (3–8).

Similarly, the evolution of external beam radiation therapy has considerably improved over the past three decades. Conventional two-dimensional planning with x-rays was shifted to three-dimensional conformal radiation therapy (3DCRT), intensity-modulated radiation therapy (IMRT), and volume arc modulated radiation therapy (VAMRT). For patients treated with IMRT or VAMART, the treatment outcomes were reported to be superior to those treated by conventional 3DCRT (9–12).

In 2016, the Prostate Testing for Cancer and Treatment (ProtecT) trial showed that the incidence rates of disease progression and metastases were lower in the radical prostatectomy and RT groups than they were in the active monitoring group. Otherwise, no significant differences existed in cancer-specific mortality among treatments (13). Since then, several studies have evaluated the clinical outcomes of patients with clinical localized prostate cancer who received different treatment modalities (14–17).

The cancer registry database in Taipei City Hospital Renai Branch was established in 2008. All patients diagnosed with prostate cancer were recruited into the database. Treatment of prostate cancer mainly follows the National Comprehensive Cancer Network (NCCN) and European Association of Urology (EAU) guidelines (18, 19). The primary aim of this study was to report oncological outcomes between IMRT/VAMRT and LRP in patients with clinically localized prostate cancer in Taiwan. The secondary aim was to document the prognostic significance of Gleason grade grouping in patients with localized prostate cancer who received IMRT/VAMRT or LRP.

## Materials and methods

The cancer registry database from Taipei City Hospital Renai Branch was queried for patients with clinically localized prostate cancer treated between January 2008 and December 2017. We excluded those with distal metastasis or clinical lymphadenopathy. Those treated with observation, androgen deprivation therapy only, open radical prostatectomy, robotic-assisted radical prostatectomy, conventional(2D) radiotherapy were also excluded from this study. A total of 218 patients were enrolled into this study. All patients received a digital rectal examination and prostate-specific antigen (PSA) checkup, followed by transrectal ultrasound biopsy of the prostate for tissue proof. All pathological reports were reviewed by two pathologists and were available for comparison. All patients underwent preoperative computed tomography (CT)/magnetic resonance imaging of the prostate and a whole-body bone scan for clinical staging. For patients who received laparoscopic radical prostatectomy, an extraperitoneal approach was applied in all patients. Surgical techniques were described in relevant studies (20, 21). For patients who received IMRT/VAMRT, pretreatment planning with CT was performed. A median radiation dose of 7,800 Gy was administered to all patients with a modulated CT follow-up on a weekly basis throughout the whole treatment period. The follow-up protocol was conducted as suggested in NCCN and EAU guidelines. The follow-up started on the date of surgery for LRP or start date of RT. Crude survival (CS) was defined by the presence of a patient's death from any possible cause. Prostate cancer-specific survival (PCSS) was defined by the presence of prostate cancer as the primary cause of death upon a patient's death in the cancer registry database. Metastasis-free survival (MFS) was defined as the time from treatment to the first detection of distant metastasis on imaging or death. Subgroup analyses were stratified by Gleason score, clinical stage, and risk group. Patients with prostate cancer in the intermediate-risk group were defined as those having a serum PSA level of 10.2–20.0 ng/ml, a clinical stage of T2b-c, and a Gleason score of 3 + 4 from pathological specimens. Patients with prostate cancer in the high-risk group were defined as those having a serum PSA level of >20.0 ng/ml, tumor extending outside the prostate, and a Gleason score of 8–10 from pathological specimens. The high-risk group was defined as having one risk factor, whereas the very-high-risk group referred to clinical stage T3b-4 and multiple biopsy samples with high-grade prostate cancer. A minimum follow-up of 12-months was required in this study.

SPSS version 25 (IBM, United States) was used for statistical analysis. The chi-square test was performed to evaluate differences between study groups, Kaplan–Meier curves were calculated for survival analyses, and statistical significance between factors was determined using the log-rank test. A P value less than 0.05 was considered statistically significant.

## Results

### Patient characteristics

From January 2008 through December 2017, 218 patients with clinically localized prostate cancer received either RT or LRP in our hospital. Of these 218 patients, 64 underwent LRP (29.4%) and 154 received RT (70.6%). The mean ages of the LRP and RT groups were 62.2 and 75.6 years, respectively (*P* < 0.01). The patients with clinical stage 2 accounted for 87% of all cases and accounted for 84% in LRP group and 87% in RT group. Gleason sum 7 and above accounted for the majority of the study population. The median follow-up periods for the LRP and RT groups were 53.5 and 64 months, respectively (maximum, 123 months in LRP and 132 months in RT). Table 1 presents demographic data.

**Table 1:** Demographic data.

	LRP group (*n* = 64)	RT group (*n* = 154)	*P*-value
Age (years)	62.17 ± 5.43	75.56 ± 6.61	<0.01
Clinical stage			
Stage 1	10	19	
Stage 2	54	135	
Gleason sum			
Gleason 4–6	11	24	
Gleason 7	29	60	
Gleason 8–10	24	70	
Median followup period (months) (range)	53.5(12–123)	64(11–132)	

LRP, laparoscopic/robotic radical prostatectomy; RT, radiotherapy*.*

### Crude survival

**Figure 1:**
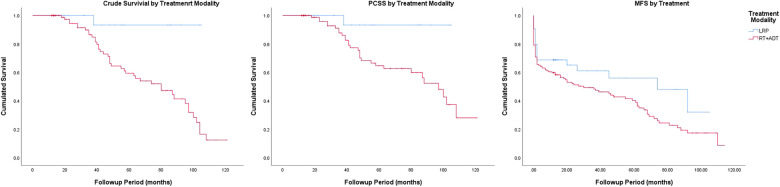
Crude survival, prostate cancer-specific survival and metastasis-free survival by treatment modality.

**Figure 2:**
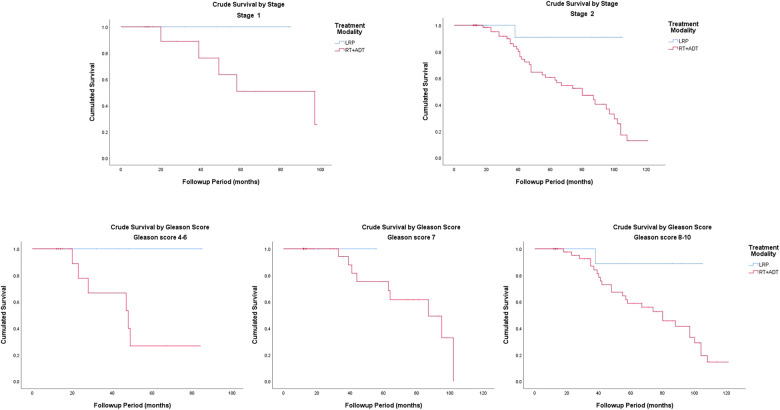
Crude survival by stage and Gleason score.

The Kaplan–Meier curves for crude survival are presented in Figure 1. The 5-year CS rate was 93.3% in the LRP group and 59.3% in the RT group. For patients with stage-1 disease, the 5-year CS rate was 100% in the LRP group and 50.8% in the RT group. For patients with stage-2 disease, the 5-year CS rate was 90.9% in the LRP group and 60.6% in the RT group. In the subgroup analysis based on a stratification by Gleason score, the 5-year CS rates were higher in the LRP group than in the RT group (*P* = 0.003). The detailed data are presented in Figure 2 and Table 2.

**Table 2:** Five-year crude survival rate, prostate cancer-specific survival, and metastasis-free survival according to treatment modalities.

Treatment	LRP (*n* = 64)	RT (*n* = 154)	*P-*value
5-year crude survival rate	93.3%	59.3%	0.004
Stage 1	100%	50.8%	0.005
Stage 2	90.9%	60.6%	
Gleason 4–6	100%	26.7%	0.003
Gleason 7	100%	75.3%	
Gleason 8–10	88.9%	41.5%	
Low risk group	100%	39.3%	0.004
Intermediate risk group	100%	79.0%	
High risk group	88.9%	58.2%	
5-year PCSS	93.3%	64.7%	0.022
Stage 1	100%	57.1%	0.027
Stage 2	90.9%	65.9%	
Gleason 4–6	100%	28.6%	0.016
Gleason 7	–%	81.3%	
Gleason 8–10	87.5%	62.3%	
Low risk group	100%	42.3%	0.02
Intermediate risk group	–%	86.2%	
High risk group	88.9%	63.7%	
5-year MFS	48.0%	40.2%	0.045
Stage 1	80.0%	26.6%	0.05
Stage 2	49.0%	42.2%	
Gleason 4–6	72.7%	8.4%	0.028
Gleason 7	65.5%	37.3%	
Gleason 8–10	48.6%	53.5%	
Low risk group	78.6%	21.3%	0.04
Intermediate risk group	61.5%	36.0%	
High risk group	48.6%	53.4%	

PCSS, prostate cancer specific survival; MFS, metastasis-free survival; LRP, laparoscopic/robotic radical prostatectomy; RT, radiotherapy.

### Prostate cancer-specific survival

**Figure 3:**
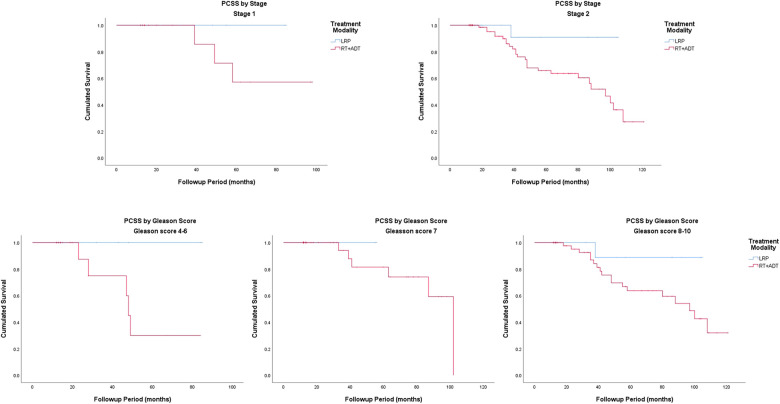
Prostate cancer-specific survival by stage and Gleason score.

The Kaplan–Meier curves for PCSS are presented in Figure 1. The 5-year PCSS rates for the LRP and RT groups were 93.3% and 64.7%, respectively. In the subgroup analysis based on a clinical stage stratification (stages 1 and 2), we found a significant difference between the LRP and RT groups (Figure 3). Furthermore, survival rates revealed similar results between the two treatment modalities when we stratified patients by the Gleason score (Table 2).

### Metastasis-free survival

**Figure 4:**
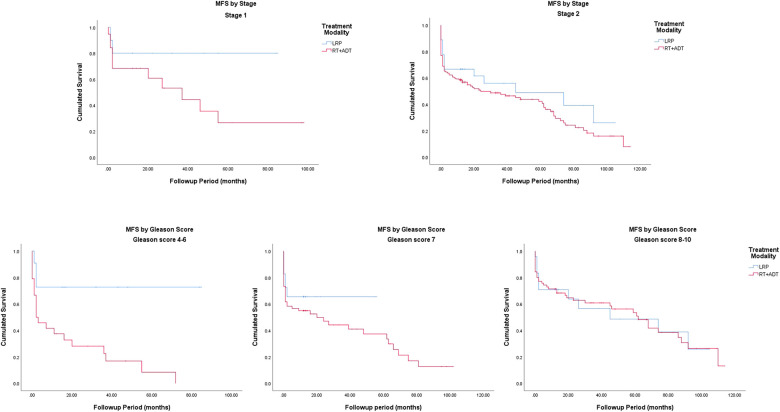
Metastasis-free survival by stage and Gleason score.

The Kaplan–Meier curves for metastasis-free survival are presented in Figure. 1. The 5-year MFS rates were 48.0% for the LRP group and 40.2% for the RT group (*P* = 0.045). In the subgroup analysis stratified by the Gleason score, a more significant survival benefit was found in the LRP group compared with the RT group (Figure 4 and Table 2).

### Survival analysis in patients with prostate cancer stratified by the risk group

The 5-year CS for patients with low-risk prostate cancer was 29.7%; that for intermediate-risk prostate cancer was 15.3%; and that for high-risk prostate cancer was 41.3%. The 5-year PCSS in the LRP group was 100% among low-risk groups, whereas it was 88.9% in high-risk groups. The 5-year PCSS in the RT group was 42.3% in patients with low-risk prostate cancer, 86.2% in patients with intermediate-risk prostate cancer, and 63.7% in patients with high-risk prostate cancer. In patients with low-risk prostate cancer, the 5-year MFS was 78.6% in the LRP group and 21.3% in the RT group. In patients with intermediate-risk prostate cancer, the 5-year MFS was 61.5% in the LRP group and 36.0% in the RT group. In patients with high-risk prostate cancer, the 5-year MFS was 48.6% in the LRP group and 53.4% in the RT group (*P* = 0.04).

## Discussion

Over the past three decades, the management of localized prostate cancer has greatly advanced, including in the introduction of the laparoscopic/robotic approach, advancement in the dose adjustment of radiation therapy, and the adequacy of active surveillance in patients with localized prostate cancer. However, the cornerstone for clinical decisions rests mainly on clinicians' judgement and patients' preferences. With improvements in various treatment modalities, it is necessary to investigate outcomes with different modalities to potentially provide an enhanced tool for clinicians in planning treatment strategies.

In 2016, Hamdy et al. (13) reported a direct comparison between various treatment modalities for the management of localized prostate cancer. They found no significant differences in PCSS among radical prostatectomy, external-beam radiotherapy, and active monitoring in the treatment of clinically localized prostate cancer, whereas surgery and RT were associated with lower incidence rates of disease progression and metastases compared with active monitoring. In the present study, we found a survival advantage in the LRP group compared with the RT group when it came to crude survival, PCSS, and MFS between the two groups.

For patients with clinically localized prostate cancer, treatment using IMRT/VAMRT has been applied for the last decade. Although short-term results have been comparable to those of radical surgery, Ma et al. (22) reported a better cancer-specific survival benefit on radical surgery compared with external-beam radiotherapy and/or brachytherapy in high-risk prostate cancer. In our study, the LRP group had a more significant survival benefit compared with the RT group.

In 2018, the average life expectancy of men in Taiwan was 77.5 years (23). In a retrospective study by Wu et al. (24), 581 patients with locally confined prostate cancer were treated with radical definitive RT in Taiwan. The researchers disclosed that they observed no differences in outcomes or toxicities in older patients with the exception of overall survival (older group: aged over 80 years). Moreover, radical prostatectomy was associated with a higher risk of postoperative sexual dysfunction and urinary incontinence in men with localized prostate cancer than was RT (25). A postal questionnaire survey conducted in Taiwan by Lin et al. (26) revealed superior urinary functions in patients who received brachytherapy compared with those who received prostatectomy. These postoperative complications affected patients' quality of life and were also a crucial factor in the selection of treatment modalities. The means of the reported 5-year CS and PCSS rates in our RT group were lower than published data (13), which might have resulted from the advanced age of patients in our RT group.

A meta-analysis by Chen et al. (27) enrolled 12 studies with 17,137 patients with localized prostate cancer and indicated that radical prostatectomy was associated with a decreased risk of overall and cancer-specific mortality compared with external beam radiotherapy. In our study, the 5-year-PCSS of the RT group reached 64.7% and even 63.7% in the high-risk group. Although LRP engendered significant survival benefit compared with RT, RT may still have a role in the management of localized prostate cancer with an estimated 5-year PCSS of more than 60%. In selective patients who cannot tolerate LRP, IMRT/VAMRT might be a strong alternative.

To our knowledge, this is the first study to provide long-term outcomes comparing LRP with RT in the treatment of localized prostate cancer in Taiwan. This study has several limitations. This was a retrospective study, and participants were recruited from a single institution. The baseline characteristics of 218 men are described in Table 1 and showed that 43.1% (*n* = 94) of them had ISUP grade group 4–5, indicating that more than one-third of patients in our study had high-risk disease. This led to a perception that clinical outcomes were mainly driven by the high-risk nature of the cohort. Less than one-fifth population was Gleason 4–6 and a worse 5-year PCSS and MFS might be associated with small amount of population in the low risk group. Although the sample size was small, we could identify the treatment benefit in patients who received radical surgery compared with radiation therapy. Hence, a prospective study on a larger scale might be required to adequately elucidate the benefit between LRP and RT groups in the future.

## Conclusions

Our results suggested that LRP provides superior long-term survival outcomes compared with RT in patients with localized prostate cancer in Taiwan. Although LRP provides favourable outcomes, clinicians can provide IMRT/VAMRT to patients considered at a high risk of perioperative/postoperative morbidity and mortality. This study represents the only study of clinically localized prostate cancer to compare LRP and IMRT/VAMRT with a 10-year follow-up.

## Data Availability

The original contributions presented in the study are included in the article/Suplementary Material, further inquiries can be directed to the corresponding author/s.
